# Predictive value of ferroptosis-related biomarkers for diabetic kidney disease: a prospective observational study

**DOI:** 10.1007/s00592-022-02028-1

**Published:** 2023-01-12

**Authors:** You Wu, Yunwei Sun, Yiwei Wu, Kecheng Zhang, Yan Chen

**Affiliations:** Department of Endocrinology, The Second Hospital of Jilin University, No. 218 Ziqiang Street, Nanguan Distract, Changchun, 130041 China

**Keywords:** Ferroptosis-related biomarkers, Risk factors, Type 2 diabetes mellitus, Kidney disease, Prospective observational study

## Abstract

**Aims:**

To explore the predictive value of ferroptosis-related (FR) biomarkers for diabetic kidney disease (DKD) in patients with type 2 diabetes mellitus (T2DM).

**Methods:**

This prospective observational study enrolled patients with T2DM at the Second Hospital of Jilin University between December 2021 and March 2022. DKD was measured by the urinary albumin-to-creatinine ratio. Receiver operating characteristic curve (ROC) analysis was performed to assess the predictive value of ferroptosis-related biomarkers for DKD.The risk factors for massive proteinuria were performed by multivariable logistic regression analysis.

**Results:**

Finally, 118 patients (53.0 ± 12.2 years, 76 males) were enrolled, 52 of them without DKD (had normal proteinuria), while 66 with DKD. (Forty-one had microproteinuria, and 25 had massive proteinuria.) FR biomarkers, including acyl-CoA synthase long chain family member 4 (ACSL4), malondialdehyde (MDA), and reactive oxygen species (ROS), were significantly higher in the massive proteinuria group than in the other groups, while glutathione peroxidase 4 (GPX4) was significantly lower (all P < 0.05). The area under the ROC of the combination of GPX4, ACSL4, MDA, and ROS for predicting DKD was 0.804 (P < 0.001). Additionally, multivariate logistic regression analysis showed that the course of disease and ferritin levels were independent risk factors for massive proteinuria, while high serum iron, transferrin, and GPX4 levels were independent protective factors for massive proteinuria in patients with T2DM (all P < 0.05).

**Conclusions:**

The GPX4, ACSL4, MDA, and ROS combination might have a good predictive value for DKD. Additionally, the course of disease, ferritin levels, serum iron, transferrin, and GPX4 were independently associated with massive proteinuria.

## Introduction

Type 2 diabetes mellitus (T2DM) is a common endocrine disorder characterized by variable degrees of insulin resistance and deficiency, resulting in hyperglycemia [1]. T2DM is one of the most common chronic diseases and can lead to life-threatening complications and reduce life expectancy [2, 3]. The 10th edition of the Diabetes Map predicts that 783 million people worldwide will have T2DM in 2045, and China will reach 174 million patients [4]. Potential complications of T2DM include cardiovascular disease, neuropathy, nephropathy, retinopathy, and increased mortality [1, 5].

Kidney disease (KD) is one of the microvascular complications of diabetes, in which the diabetic kidney gradually enters the stage of chronic kidney disease (CKD) [6, 7]. The timely diagnosis and treatment of KD in diabetes (DKD) can delay its progression and the occurrence of complications, but a study in 2021 showed that about half of the patients with T2DM are already in CKD stage 3 when diagnosed [8]. Therefore, specific biomarkers for DKD are being sought, but the search has remained unsuccessful. In addition, there are differences between animal models used in preclinical studies of DKD and clinical studies regarding age, renal function at onset, and combination medication. These differences lead to the poor predictive value of animal experiments for the results of clinical trials. DKD is difficult to treat, and controlling DKD progression is not ideal [9]. Various phenotypes of DKD increase the difficulties for early diagnosis and intervention [10], and new biomarkers are necessary.

Ferroptosis was initially found to be involved in the development of ischemia–reperfusion injury, stroke, and cancer [11, 12], but there are relatively few studies on the role of ferroptosis in T2DM and its complications. Like other regulatory cell death pathways, ferroptosis is controlled by a complex regulatory network [11, 12]. Intervention on any pathway’s members can have a therapeutic effect on diseases. Ferroptosis inhibitors were first observed to improve β-cell damage in pancreatic islets in vitro [13], and the role of ferroptosis was subsequently found in diabetic complications, such as diabetic cardiomyopathy and diabetic retinopathy [14, 15]. For DKD, the iron levels in the kidneys of diabetic rats and the urine of diabetic patients are elevated, and a low-iron diet or iron chelators can delay the development of DKD in diabetic patients [16], which is indirect evidence of the involvement of ferroptosis in the pathogenesis of DKD. In the process of lipid peroxidation, the increased acyl-CoA synthase long chain family member 4 (ACSL4) activity increases the sensitivity of the cell membrane to ferroptosis [17]. The iron in the unstable iron pool simultaneously generates a large amount of reactive oxygen species (ROS) that participate in peroxidation and ferroptosis [18, 19]. On the other hand, glutathione peroxidase 4 (GPX4) prevents ferroptosis [20].

Therefore, this study aimed to explore the predictive value of ferroptosis-related (FR) biomarkers for diabetes kidney disease (DKD) in patients with type 2 diabetes mellitus (T2DM).

## Methods

### Study design and participants

This prospective observational study enrolled patients with T2DM at the Second Hospital of Jilin University between December 2021 and March 2022. This study was approved by the Ethics Committee of the Second Hospital of Jilin University. All participants signed the informed consent form.

The inclusion criteria were (1) 18–75 years of age, (2) the diagnosis of T2DM conformed to the “Chinese Guidelines for the Prevention and Treatment of Type 2 Diabetes” (2020 edition) [21]. The exclusion criteria were (1) type 1 diabetes mellitus, monogenic diabetes syndrome, or other special types of diabetes, (2) acute complications of diabetes or other serious complications, (3) combined with severe liver, kidney, or heart disease, (4) anemia or used iron treatment in the past 3 months, or (5) tumor, inflammation, infection, or any disease that could affect serum iron metabolism-related biomarkers.

### Data collection and definitions

The demographic clinical data of the patients, such as age, sex, course of disease, height, and weight, were collected. Body mass index (BMI) was calculated as BMI = weight (kg)/m^2^. Venous blood was collected after 8 h of fasting to measure the general biochemical biomarkers. Glycated hemoglobin (HbA1C) was determined by high-performance liquid chromatography (HLC-723G8). An automatic biochemical analyzer (UniCel DxC 800 Synchron) was used to determine fasting plasma glucose (FPG), triglycerides (TG), total cholesterol (TC), high-density lipoprotein cholesterol (HDL-C), low-density lipoprotein cholesterol (LDL-C), alanine aminotransferase (ALT), aspartate aminotransferase (AST), blood urea nitrogen (BUN), serum creatinine (Scr), cystatin C (Cys-C), and serum iron metabolism related biomarkers (serum iron, ferritin, and transferrin). The estimated glomerular filtration rate (eGFR) was calculated: female eGFR = 130 × (Scr/62)^a^ × (Cys-C/0.80)^b^ × (0.995)^age^ [age ≤ 62 years, *a* = − 0.248; age > 62, *a* = − 0.601; Cys-C ≤ 0.08 mg/L, *b* = − 0.375; Cys-C > 0.08 mg/L, *b* = − 0.711]; male eGFR = 135 × (Scr/80)^a^ × (Cys-C/0.80)^b^ × (0.995)^age^ [age ≤ 80, *a* = − 0.207; age > 62, *a* = − 0.601; Cys-C ≤ 0.08 mg/L, *b* = − 0.375, Cys-C > 0.08 mg/L, *b* = − 0.711].

The UACR was measured using an IMMAGE 800 analyzer. According to the diagnosis of DKD conformed to the “Chinese Guidelines for the Prevention and Treatment of Diabetic Kidney Diseases” (2021 edition) [22], patients were divided into non-DKD and DKD group. Patients in non-DKD group had normal proteinuria (the urinary albumin to creatinine ratio [UACR] < 30 mg/g), while in DKD group were divided into microproteinuria subgroup (UACR 30–300 mg/g) and massive proteinuria subgroup (UACR > 300 mg/g).

#### Methods for the detection of ferroptosis-related biomarkers

Cubital venous blood (2 mL) was collected from the patients after fasting for 8–12 h in the morning. After standing at room temperature for 30 min, the samples were centrifuged at 3500 rpm for 10–15 min. The serum samples were collected and stored at − 80 °C. The levels of the ferroptosis-related biomarkers GPX4, ACSL4, ROS, and MDA were detected by enzyme-linked immunosorbent assay kits (OUSAID, Hunan, China); the lowest detection concentrations were 3.75 ng/mL, 33.75 pg/mL, 15 IU/mL, and 0.15 nmol/mL, respectively. Antibody specific for Human ferroptosis-related biomarkers (GPX4, ACSL4, MDA, and ROS) has been pre-coated onto a microplate. Standard, samples and HRP-linked detect antibody specific for ferroptosis-related biomarkers are pipetted into the wells and ferroptosis-related biomarkers present is bound by the immobilized antibody and detect antibody following incubation. After washing away any unbound substances, streptavidin-HRP is added. After washing, substrate solution is added to the wells and color develops in proportion to the amounts of ferroptosis-related biomarkers bound in the initial step. The color development is stopped and the intensity of the color is measured.

### Statistical analysis

SPSS 26.0 statistical software (IBM Corp., Armonk, NY, USA) was used for data analysis. The continuous data that conformed to the normal distribution (according to the Kolmogorov–Smirnov test) were described as means ± standard deviation (SD) and analyzed using ANOVA. Continuous data with a skewed distribution were described as medians (upper and lower quartiles) and analyzed using the Kruskal–Wallis H-test. Categorical data were expressed as n (%) and analyzed using the chi-square test. The Pearson correlation analysis was used to analyze the correlations of continuous data that conformed to the normal distribution, while the Spearman correlation analysis was used to analyze the correlations between categorical data and continuous data with a skewed distribution. Receiver operating characteristics (ROC) curves were drawn using GraphPad Prism 9 (GraphPad Software Inc., San Diego, CA, USA), and the area under the curve (AUC) was calculated to evaluate the predictive value of ferroptosis-related biomarkers for DKD. Sensitivity, specificity, cutoff value, positive predictive value (PPV) and negative predictive value (NPV) were calculated by using MedCalc statistical software (MedCalc Software Ltd, Ostend, Belgium). The risk factors for massive proteinuria were performed by multivariable logistic regression analysis. Two-sided *P* values < 0.05 were considered statistically significant.

## Results

**Fig. 1:**
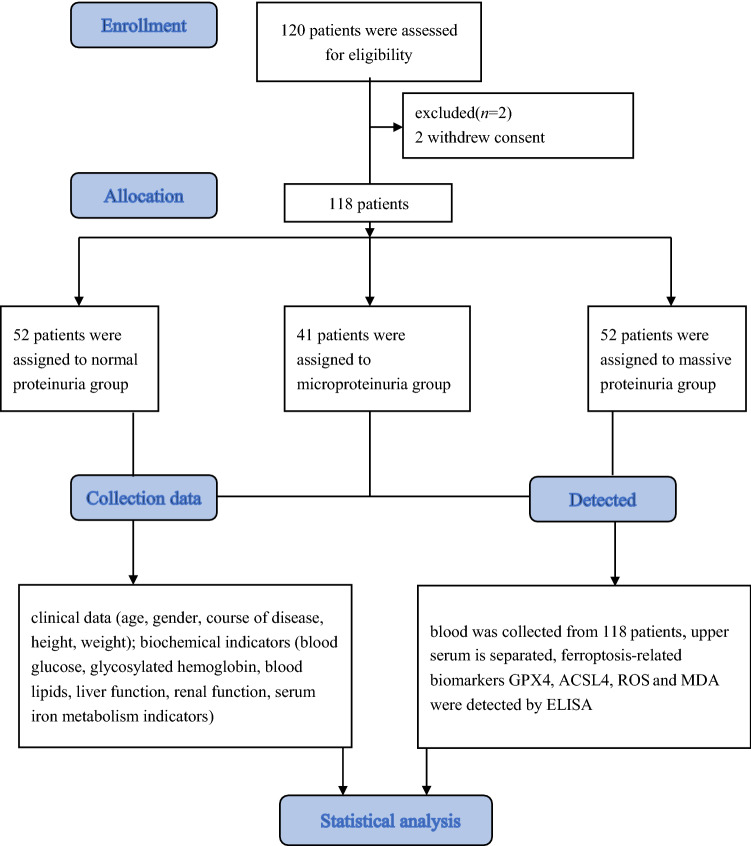
Study flowchart

The participant flowchart is depicted in Fig. 1. Finally, 118 participants were enrolled, with an average age of 53.0 ± 12.2 years and including 76 males and 42 females. Among them, the course of the disease, HbA1C, and ALT in the normal proteinuria group were lower than in the massive proteinuria group (all *P* < 0.05). eGFR and serum iron in the massive proteinuria group were lower than in the other two groups (all *P* < 0.05). The levels of ACSL4, MDA, and ROS in the massive proteinuria group were higher than in the other groups (all *P* < 0.05). UACR, transferrin, and GPX4 levels gradually decreased with the aggravation of proteinuria, while ferritin levels gradually increased (all *P* < 0.05) (Table 1).

**Table 1:** Characteristics of the patients

Variables	Non-DKD (*n* = 52)	DKD (*n* = 66)		*P*
	Normal proteinuria (*n* = 52)	Microproteinuria (*n* = 41)	Massive proteinuria (*n* = 25)	
Age	51.8 ± 10.36	51.5 ± 12.8	56.2 ± 14.4	0.320
Sex (%)				
Male	37 (71.2%)	23 (56.1%)	16 (64.0%)	0.314
Female	15 (28.8%)	18 (43.9%)	9 (36.0%)	
Height (cm)	168.6 ± 7.9	168.0 ± 9.8	163.2 ± 21.3	0.190
Weight (kg)	75.9 ± 13.8	77.8 ± 15.5	73.5 ± 11.5	0.467
BMI (kg/m^2^)	26.6 ± 3.4	27.6 ± 4.2	26.2 ± 2.9	0.247
Course of disease (years)	5.0 (1.0, 10.0)	7.0 (2.0, 11.0)	10.0 (5.5, 16.5)	**0.021**^**c**^
FPG (mmol/L)	9.34 ± 3.07	9.90 ± 3.54	10.24 ± 3.52	0.501
HbA1C (%)	7.88 ± 1.22	8.52 ± 1.78	9.16 ± 1.16	** < 0.001**^**c**^
ALT (U/L)	29.00 (21.00, 45.00)	24.00 (17.00, 48.00)	19.00 (15.00, 33.50)	**0.033**^**c**^
AST (U/L)	22.00 (16.00, 25.75)	19.00 (17.00.27.50)	18.00 (14.50, 25.50)	0.183
TG (mmol/L)	2.19 (1.44, 3.10)	2.53 (1.83, 3.64)	2.23 (1.16, 3.12)	0.239
TC (mmol/L)	5.68 ± 1.01	5.83 ± 1.63	5.54 ± 1.57	0.773
HDL-C (mmol/L)	1.06 (0.93, 1.28)	1.14 (0.95, 1.31)	1.04 (0.86, 1.21)	0.314
LDL-C (mmol/L)	3.11 ± 0.87	2.87 ± 1.13	2.97 ± 1.37	0.540
BUN (mmol/L)	5.06 (3.93, 6.04)	5.30 (4.32, 6.04)	5.52 (4, 82, 7.93)	0.079
Scr (μmol/L)	71.00 (62.25, 78.75)	75.00 (63.00, 87.00)	81.00 (62.50, 90.00)	0.057
UACR (mg/g)	9.86 (5.44, 16.00)	87.69 (45.66, 121.51)	676.26 (479.26, 1241.63)	** < 0.001**^**d**^
eGFR (ml/min)	98.35 ± 12.75	94.34 ± 23.48	79.06 ± 32.01	**0.018**^**bc**^
Iron (µmol/L)	21.44 ± 5.55	19.03 ± 6.15	14.42 ± 3.21	** < 0.001**^**bc**^
Ferritin (µg/L)	117.00 (85.75, 175.75)	203.00 (138.00, 237.50)	378.00 (319.00, 439.50)	** < 0.001**^**d**^
Transferrin (g/L)	2.44 ± 0.35	2.04 ± 0.35	1.46 ± 0.69	** < 0.001**^**d**^
GPX4 (ng/mL)	164.09 ± 29.80	145.55 ± 25.38	119.70 ± 23.73	** < 0.001**^**d**^
ACSL4 (pg/mL)	1249.99 ± 343.90	1368.30 ± 347.54	1602.55 ± 268.45	** < 0.001**^**bc**^
MDA (nmol/mL)	7.43 ± 1.29	7.80 ± 1.96	9.48 ± 1.35	** < 0.001**^**bc**^
ROS (IU/mL)	416.82 ± 117.90	440.22 ± 136.23	498.81 ± 108.69	**0.026**^**bc**^

^a^There was a statistical difference between the normal proteinuria group and the microproteinuria group (*P* < 0.05)

^b^There was a statistical difference between the microproteinuria group and the massive proteinuria group (*P* < 0.05)

^c^There was a statistical difference between the normal proteinuria group and the massive proteinuria group (*P* < 0.05)

^d^The data in the three groups were all statistically different (*P* < 0.05)

*BMI* body mass index, *FPG* fasting plasma glucose, *HbA1C* glycated hemoglobin, *ALT* alanine aminotransferase, *AST* aspartate aminotransferase, *TG* triglycerides, *TC* total cholesterol, *HDL-C* high-density lipoprotein cholesterol, *LDL-C* low-density lipoprotein cholesterol, *BUN* blood urea nitrogen, *Scr* serum creatinine, *UACR* urine albumin-creatinine ratio, *eGFR* estimated glomerular filtration rate, *MDA* malondialdehyde, *ROS* reactive oxygen species

**Fig. 2:**
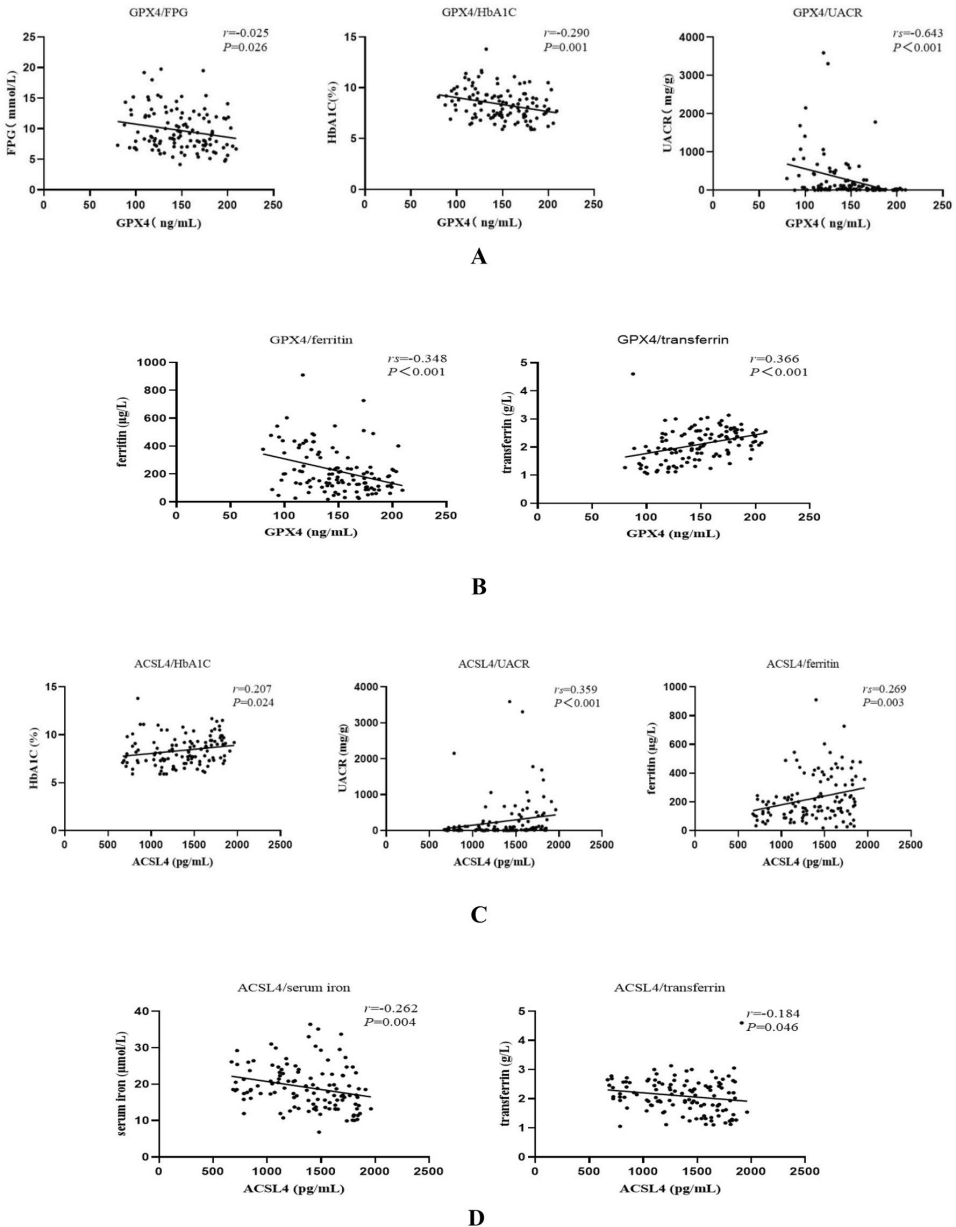

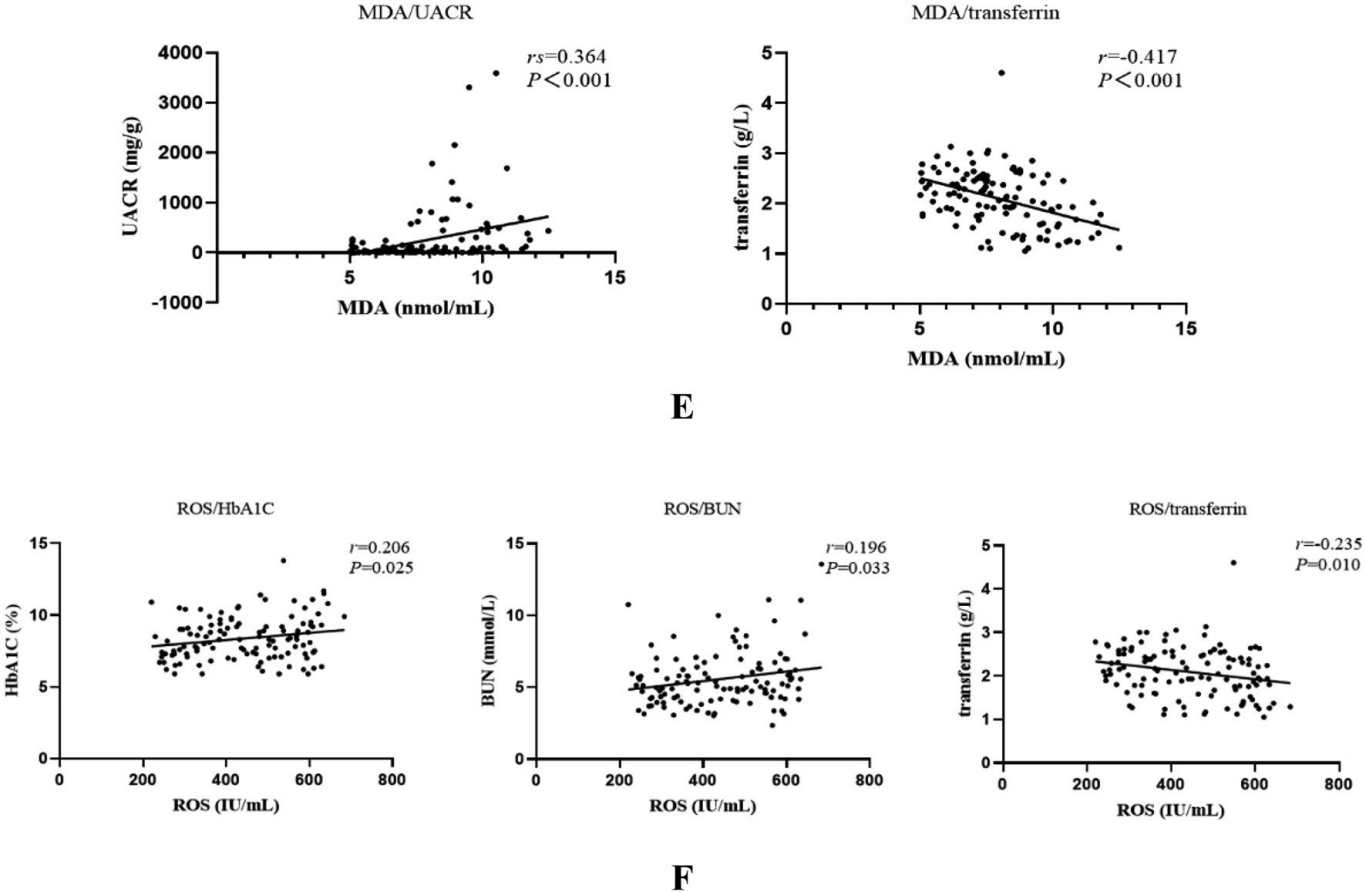
**A** Correlation analysis of GPX4 with fasting plasma glucose (FPG), glycated hemoglobin (HbA1C), and urine albumin to creatinine ratio (UACR). **B** Correlation analysis of GPX4 with ferritin and transferrin. **C** Correlation analysis of ACSL4 with HbA1C, urine UACR, and ferritin. **D** Correlation analysis of ACSL4 with serum iron and transferrin. **E** Correlation analysis of malondialdehyde (MDA) with UACR and transferrin. **F** Correlation analysis of reactive oxygen species (ROS) with HbA1C, blood urea nitrogen (BUN), and transferrin

GPX4 was negatively correlated with FPG, HbA1C, UACR, and ferritin and positively correlated with transferrin (*r* = − 0.205, *r* = − 0.290, *r*_s_ = − 0.463, *r*_s_ = − 0.348, and *r* = 0.366, respectively; all *P* < 0.05) (Fig. 2A, B, Table 2). ACSL4 was positively correlated with HbA1C, UACR, and ferritin and negatively correlated with serum iron and transferrin (*r* = 0.207, *r*_s_ = 0.359, *r*_s_ = 0.269, *r* = − 0.262, and *r* = − 0.184, respectively; all *P* < 0.05) (Fig. 2C, D, Table 2). MDA was positively correlated with UACR and negatively correlated with transferrin (*r*_s_ = 0.364 and *r* = − 0.417, respectively; both *P* < 0.05) (Fig. 2E, Table 2). ROS was positively correlated with HbA1C and BUN and negatively correlated with transferrin (*r* = 0.206, *r*_s_ = 0.196, and *r* = − 0.235, respectively; all *P* < 0.05) (Fig. 2F, Table 2).

**Table 2:** Correlation analysis of ferroptosis-related biomarkers and clinical biomarkers

Variables	GPX4		ACSL4		MDA		ROS	
	*r*/*r*_s_	*P*	*r*/*r*_s_	*P*	*r*/*r*_s_	*P*	*r*/*r*_s_	*P*
Age	− 0.037	0.692	− 0.076	0.412	− 0.031	0.742	0.125	0.176
Sex	0.070	0.450	0.050	0.595	− 0.017	0.852	− 0.017	0.858
Height (cm)	− 0.020	0.827	− 0.066	0.475	− 0.062	0.502	0.008	0.932
Weight (kg)	− 0.125	0.178	0.019	0.839	0.099	0.286	0.005	0.956
BMI (kg/m^2^)	− 0.068	0.463	0.054	0.565	0.103	0.267	0.022	0.810
Course of disease (years)	− 0.084	0.364	0.132	0.153	0.166	0.072	0.051	0.582
FPG (mmol/L)	− 0.205	**0.026**	0.093	0.318	− 0.005	0.956	0.103	0.266
HbA1C (%)	− 0.290	**0.001**	0.207	**0.024**	0.111	0.231	0.206	**0.025**
ALT (U/L)	0.004	0.968	− 0.163	0.079	− 0.086	0.357	− 0.035	0.709
AST (U/L)	0.024	0.795	− 0.130	0.160	− 0.058	0.535	− 0.027	0.770
TG (mmol/L)	− 0.093	0.319	− 0.016	0.862	− 0.062	0.503	− 0.114	0.220
TC (mmol/L)	0.057	0.541	− 0.004	0.965	− 0.096	0.303	− 0.049	0.599
HDL-C (mmol/L)	0.098	0.293	− 0.022	0.814	− 0.056	0.546	− 0.055	0.557
LDL-C (mmol/L)	0.027	0.774	− 0.038	0.685	− 0.017	0.858	− 0.006	0.946
BUN (mmol/L)	− 0.131	0.158	0.070	0.451	− 0.040	0.664	0.196	**0.033**
Scr (μmol/L)	− 0.037	0.692	0.100	0.280	0.006	0.945	− 0.055	0.552
UACR (mg/g)	− 0.463	** < 0.001**	0.359	** < 0.001**	0.364	** < 0.001**	0.127	0.172
eGFR (ml/min)	0.074	0.425	− 0.156	0.092	− 0.047	0.617	− 0.085	0.359
Iron (µmol/L)	0.159	0.085	− 0.262	**0.004**	− 0.128	0.169	− 0.039	0.679
Ferritin (µg/L)	− 0.348	** < 0.001**	0.269	**0.003**	0.143	0.122	0.056	0.549
Transferrin (g/L)	0.366	** < 0.001**	− 0.184	**0.046**	− 0.417	** < 0.001**	− 0.235	**0.010**

The bold means that the P value is less than 0.05

*BMI* body mass index, *FPG* fasting plasma glucose, *HbA1C* glycated hemoglobin, *ALT* alanine aminotransferase, *AST* aspartate aminotransferase, *TG* triglycerides, *TC* total cholesterol, *HDL-C* high-density lipoprotein cholesterol, *LDL-C* low-density lipoprotein cholesterol, *BUN* blood urea nitrogen, *Scr* serum creatinine, *UACR* urine albumin/creatinine ratio, *eGFR* estimated glomerular filtration rate, *MDA* malondialdehyde, *ROS* reactive oxygen species

**Fig. 3:**
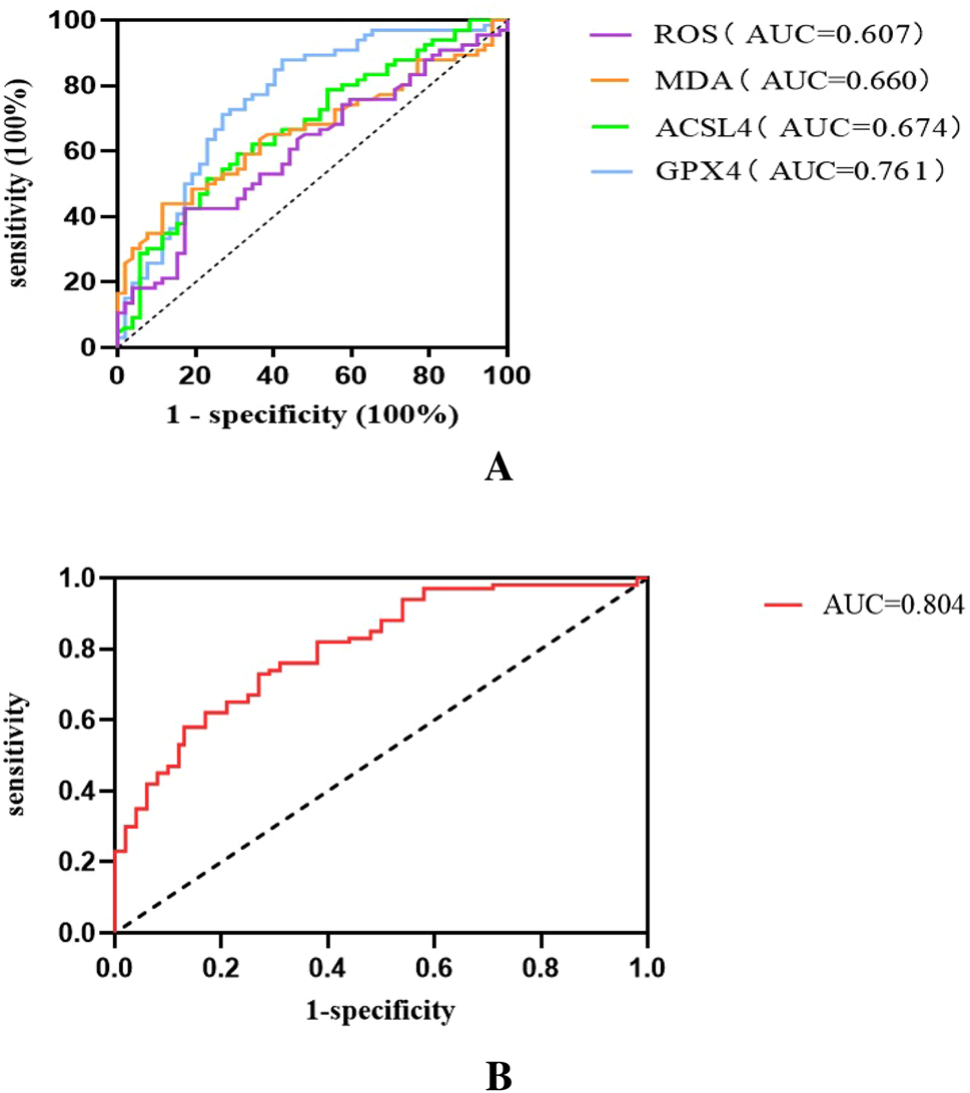
**A** Receiver operating characteristics (ROC) curve of GPX4, ACSL4, malondialdehyde (MDA), and reactive oxygen species (ROS) for predicting diabetic kidney disease. **B** ROC curve of the combination of GPX4, ACSL4, MDA, and ROS for predicting diabetic kidney disease

The microproteinuria and massive proteinuria groups were combined, and the participants were divided into the non-DKD (*n* = 52) and DKD (*n* = 66) groups. The ROC curves were drawn to explore the clinical predictive value of ferroptosis-related biomarkers on diabetic kidney disease (Fig. 3A). GPX4, ACSL4, MDA, and ROS all had a certain predictive value for the occurrence of DKD in patients with T2DM. GPX4 had the best predictive value. Using GPX4 167.5 as the best threshold displayed 88% sensitivity and 58% specificity (Table 3). The AUC of the combination of GPX4, ACSL4, MDA, and ROS for predicting DKD was 0.804 (*P* < 0.001) (Fig. 3B).

**Table 3:** Predictive value of ferroptosis-related biomarkers for diabetic kidney disease

Variables	AUC	Cut-off	Sensitivity (%)	Specificity (%)	PPV	NPV
GPX4 (ng/mL)	0.761	< 167.50	87.88	57.69	18.8	97.7
ACSL4 (pg/mL)	0.674	> 1511.39	51.52	76.92	19.9	93.5
MDA (nmol/mL)	0.660	> 8.76	43.94	88.46	29.7	93.4
ROS (IU/mL)	0.607	> 532.00	42.42	82.69	21.4	92.8

*HbA1C* glycated hemoglobin, *ALT* alanine aminotransferase, *MDA* malondialdehyde, *ROS* reactive oxygen species

Additionally, univariate logistic regression analysis showed that the course of the disease, HbA1C, serum iron, ferritin, transferrin, GPX4, ACSL4, MDA, and ROS were potential risk factors for massive proteinuria (all *P* < 0.05) (Table 4). Furthermore, multivariate logistic regression analysis suggested that the course of disease (odds ratio [OR] = 1.345, 95% CI: 1.036–1.746, *P* = 0.026) and ferritin levels (OR = 1.030, 95% CI 1.006–1.055, *P* = 0.014) were independent risk factors for massive proteinuria, while high serum iron (OR = 0.418, 95% CI 0.205–0.853, *P* = 0.017), transferrin (OR = 0.053, 95% CI 0.008–0.363, *P* = 0.003), and GPX4 (OR = 0.935, 95% CI 0.879–0.994, *P* = 0.031) levels were inversely independent protective factors for massive proteinuria in patients with T2DM (Table 4).

**Table 4:** Univariate and multivariate logistic analysis of the influence of massive proteinuria in T2DM

Variables	Univariate logistic regression		Multivariate logistic regression	
	OR, 95% CI	P	OR, 95%CI	P
Course of disease	1.111, (1.034, 1.193)	**0.004**	1.345, (1.036,1.746)	**0.026**
HbA1C (%)	1.857, (1.295, 2.665)	**0.001**	10.130, (0.990,103.651)	0.051
ALT (U/L)	0.977, (0.950, 1.004)	0.094	–	
Iron (µmol/L)	0.734, (0.638, 0.844)	** < 0.001**	0.418, (0.205,0.853)	**0.017**
Ferritin (µg/L)	1.008, (1.004, 1.012)	** < 0.001**	1.030, (1.006,1.055)	**0.014**
Transferrin (g/L)	0.003, (0.000, 0.024)	** < 0.001**	0.053, (0.008,0.363)	**0.003**
GPX4 (ng/mL)	0.941, (0.919, 0.964)	** < 0.001**	0.935, (0.879,0.994)	**0.031**
ACSL4 (pg/mL)	1.004, (1.002, 1.005)	** < 0.001**	1.004, (0.998,1.010)	0.194
MDA (nmol/mL)	2.222, (1.547, 3.192)	** < 0.001**	7.942, (0.524,120.475)	0.135
ROS (IU/mL)	1.006, (1.001, 1.010)	**0.009**	1.026, (1.992,1.061)	0.136

The bold means that the P value is less than 0.05

*AUC* area under the curve, *Cut-off* cut off value, *PPV* positive predictive value, *NPV* negative predictive value

## Discussion

The results suggest that the combination of GPX4, ACSL4, MDA, and ROS might have a good predictive value for DKD. Additionally, the course of disease, ferritin levels, serum iron, transferrin, and GPX4 were independently associated with massive proteinuria. These findings might provide a prediction model consisted of ferroptosis-related biomarkers for DKD in patients with T2DM.

Ferroptosis is regulated by multiple factors. ACSL4 activity increases the sensitivity of cell membrane to ferroptosis [17]. ROS is both a consequence and an actor of ferroptosis, and MDA is an oxidation product [18, 19]. On the other hand, GPX4 prevents ferroptosis [20]. The present study showed that the levels of ACSL4, MDA, and ROS in the massive proteinuria group were higher than in the other two groups, and the expression of GPX4 was lower than that of the other two groups, as supported by the theoretical concepts of these four molecules in ferroptosis. In addition, GPX4 was negatively correlated with UACR, while ACSL4 and MDA were positively correlated with UACR. The univariable logistic regression analyses showed that the four biomarkers were associated with massive proteinuria, but only GPX4 was an independent factor in the multivariable regression analysis. It is supported by Wang et al. [23], who also observed a significant increase in ACSL4 level, a significant decrease in GPX4 level, and an increase in the content of lipid peroxidation products in the DKD mouse model. In addition, the ACSL4 inhibitor rosiglitazone reduced the content of lipid peroxidation products [23]. In addition to animal models, decreased GPX4 expression was also found in kidney biopsies from patients with DKD [24]. Feng et al. [25] reported that ferroptosis could aggravate the renal injury and tubular fibrosis in DKD mice through the hypoxia-inducible factor-1α pathway. The glutathione system is the main pathway limiting ferroptosis [26]. The inhibition of GPX4 increases ferroptosis [27]. The conditional depletion of GPX4 in mice increases ferroptosis and leads to apoptosis, necroptosis, and pyroptosis [28–30]. These results highlight the central role of GPX4 in regulating ferroptosis, and ferroptosis is involved in the pathogenesis of DKD [31].

At present, the gold standard for the diagnosis of DKD is still renal biopsy, but because of its invasiveness, some patients can refuse or be ineligible because of comorbidities. In addition, it requires equipment, supplies, and skilled operators. In this context, screening for DKD using a simple blood draw is attractive. According to the ROC analysis, the AUC of GPX4 alone was 0.761, while adding ACSL4, MDA, and ROS increased the AUC to 0.804. Therefore, combining the above four biomarkers had a certain predictive power on DKD.

Iron homeostasis in the body is strictly regulated; when iron is in excess, highly toxic hydroxyl radicals are generated through the Fenton reaction, which induces oxidative stress [10, 17]. It has been reported that iron restriction can prevent the progression of DKD, and in DKD rats, treatment with the iron chelator deferiprone was found to reduce renal inflammatory infiltration, fibrosis, and oxidative stress and to have a protective effect on the kidneys [32]. In this study, the levels of serum iron and transferrin in the massive proteinuria group were higher than in the normal proteinuria group, while ferritin levels were higher than in the other two groups. Serum iron, transferrin, and ferritin were independently associated with massive proteinuria in DKD. In a retrospective study in China, the differences in serum iron, transferrin, and ferritin at baseline were compared between T2DM and DKD patients, and it was found that lower serum transferrin was a predictor of DKD progression to ESRD, supporting the present study [33]. Ferritin is the main form of iron storage, reflecting the body’s iron overload to a certain extent. When ferritin is saturated, excess iron enters the kidney in the form of non-transferrin-bound iron and catalyzes the production of ROS, resulting in cell damage, accompanied by increased serum ROS and MDA levels and inflammation (as shown by interleukin-6 and tumor necrosis factor-α in the kidneys), while the antioxidants such as glutathione and superoxide dismutase in the kidneys are decreased [34]. The univariable regression analysis in this study also found that HbA1C is a risk factor for massive proteinuria in DKD patients. Therefore, it is necessary to strengthen early glycemic control to reduce the decline of eGFR. However, the current HbA1C control goals are not unified. The KDIGO guidelines recommend a target range of 6.5% to 8.0% for HbA1C based on each patient’s degree of hypoglycemia risk [35]. In the natural history of DKD, hyperglycemia is the main factor driving its progression [36]. Hyperglycemia can lead to increased advanced glycation products, the activation of the polyol pathway and endothelial cell damage, vascular proliferation, and podocyte and renal tubular epithelium damage by activating downstream cytokines [36]. In addition to glycemia, the course of the disease was also independently associated with massive proteinuria in patients, supported by Wei et al. [37].

This study had limitations. The patients were from a single hospital, resulting in a small sample size. Renal biopsies could not be performed for ethical reasons, but future studies should enroll patients who undergo renal biopsy for medical reasons and examine the associations among histological changes and ferroptosis-related biomarkers. Various immune and inflammatory markers associated with the pathogenesis of DKD were not measured either. Information on whether patients had fatty liver was not collected.

In conclusion, the combination of GPX4, ACSL4, MDA, and ROS might have a good predictive value for DKD. Additionally, the course of disease, ferritin levels, serum iron, transferrin, GPX4 were independently associated with massive proteinuria. Nevertheless, the prediction model consisted of ferroptosis-related biomarkers for DKD in patients with T2DM needs validation by multicenter with large sample size.

## Data Availability

The datasets generated and/or analyzed during the current study are not publicly available due [Protect the privacy of patients] but are available from the corresponding author on reasonable request.

## Abbreviations

FR: Ferroptosis-related
DKD: Diabetic kidney disease
T2DM: Type 2 diabetes mellitus
UACR: Urinary albumin to creatinine ratio
ROC: Receiver operating characteristic curve
ACSL4: Cyl-CoA synthase long chain family member 4
MDA: Malondialdehyde
ROS: Reactive oxygen species
GPX4: Glutathione peroxidase 4
KD: Kidney disease
CKD: Chronic kidney disease
BMI: Body mass index
HbA1C: Glycated hemoglobin
FPG: Fasting plasma glucose
TG: Triglycerides
TC: Total cholesterol
HDL-C: High-density lipoprotein cholesterol
LDL-C: Low-density lipoprotein cholesterol
ALT: Alanine aminotransferase
AST: Aspartate aminotransferase
BUN: Blood urea nitrogen
Scr: Serum creatinine
Cys-C: Cystatin C
eGFR: Estimated glomerular filtration rate
AUC: Area under the curve
